# Amino Acid Catabolism During Nitrogen Limitation in *Phaeodactylum tricornutum*

**DOI:** 10.3389/fpls.2020.589026

**Published:** 2020-12-17

**Authors:** Yufang Pan, Fan Hu, Chen Yu, Chenjie Li, Teng Huang, Hanhua Hu

**Affiliations:** ^1^Key Laboratory of Algal Biology, Institute of Hydrobiology, Chinese Academy of Sciences, Wuhan, China; ^2^School of Foreign Languages, China University of Geosciences, Wuhan, China; ^3^University of Chinese Academy of Sciences, Beijing, China

**Keywords:** diatom, amino acid, catabolism, nitrogen, triacylglycerols

## Abstract

Diatoms can accumulate high levels of triacylglycerols (TAGs) under nitrogen depletion and have attracted increasing attention as a potential system for biofuel production. In *Phaeodactylum tricornutum*, a model diatom, about 40% of lipid is synthesized from the breakdown of cellular components under nitrogen starvation. Our previous studies indicated that carbon skeletons from enhanced branched-chain amino acid (BCAA) degradation under nitrogen deficiency contribute to TAG biosynthesis in *P. tricornutum*. In this review, we outlined the catabolic pathways of all 20 amino acids based on the genome, transcriptome, proteome, and metabolome data. The contribution of these amino acid catabolic pathways to TAG accumulation was also analyzed.

## Introduction

Diatoms are a group of unicellular eukaryotic algae and an important component of marine phytoplankton. Although thousands of genes were of green algal derivation (Moustafa et al., 2009), diatoms are believed to emerge as the result of a secondary endosymbiotic event between two eukaryotes, a red alga and an oomycete (Medlin et al., 2000). Therefore, diatoms possess some unique features in comparison with other photosynthetic eukaryotes, including the presence of hundreds of genes from bacteria, the Entner–Doudoroff pathway, and urea cycle unfound in plants and green algae (Bowler et al., 2008; Allen et al., 2011; Fabris et al., 2012; Singh et al., 2015). *Phaeodactylum tricornutum* is one of the model diatoms with short generation time, and routine and simple genetic manipulation is available (Zhang and Hu, 2014; Karas et al., 2015; Falciatore et al., 2020). This diatom has the capacity to accumulate eicosapentaenoic acid, fucoxanthin, and neutral lipids (mostly triacylglycerols, TAGs) and thus is perceived as a microalgal cell factory and a potential system for biofuel production (Butler et al., 2020).

Nitrogen, accounting for over 7% of cellular mass in marine microorganisms, is one of the major constituents of both proteins and nucleic acids (Geider and La Roche, 2002). Many nutrients and nitrogen in particular are restricted in the open ocean (Moore et al., 2013), though seasonal inputs of nitrate (NO_3_^–^) can cause diatom-dominated phytoplankton blooms in coastal ecosystems (Kudela and Dugdale, 2000). Diatoms are capable of assimilating dissolved nitrogen sources of different forms, including inorganic ones such as nitrate (NO_3_^–^), nitrite (NO_2_^–^), and ammonium (NH_4_^+^) and organic ones such as urea and amino acids (Jauffrais et al., 2016). Amino acids can be taken up by cells and intracellularly metabolized as diatom genomes containing plasma membrane amino acid transporters (Armbrust et al., 2004; Sipler and Bronk, 2015), and they can also be oxidized by extracellular L-amino acid oxidase to produce α-keto acid, NH_4_^+^, and hydrogen peroxide (Palenik and Morel, 1990; Rees and Allison, 2006; Contreras and Gillard, 2020). Under nitrogen stress, cellular protein content decreases and amino acid degradation occurs (Guerra et al., 2013). Acetyl-CoA, a product of the metabolism of some amino acids, enters the tricarboxylic acid (TCA) cycle and is shunted toward fatty acid biosynthesis (Hockin et al., 2012; Ge et al., 2014; Levitan et al., 2015). It is indicated that carbon skeletons from enhanced branched-chain amino acid (BCAA) degradation under nitrogen deficiency feed into the TCA cycle and contribute to TAG biosynthesis in *P. tricornutum* (Ge et al., 2014; Pan et al., 2017) and *Chlamydomonas reinhardtii* (Liang et al., 2019). However, few studies have examined the interaction between metabolic pathways of the other amino acids and TAG biosynthesis. Although amino acid biosynthesis pathways have been reviewed (Bromke, 2013), catabolic pathways of amino acids in *P. tricornutum* have not been summarized. In plants, amino acid catabolism and regulation have received considerable attention (Hildebrandt et al., 2015), and amino acid catabolism is important not only during normal senescence but also in stress tolerance. The capacity of diatoms to use dissolved amino acids has been considered to help diatoms survive in blooms or in light-impenetrable sediments (Admiraal and Peletier, 1979). In response to nitrogen deprivation, amino acid degradation could promote the redistribution of carbon and nitrogen flow in diatom cells (Alipanah et al., 2015).

In this review, we outlined the catabolic pathways of all 20 amino acids and provided the subcellular localization information of related enzymes according to the prediction (Supplementary Material) from genome annotation in *P. tricornutum*. Based on published transcriptomes (Levitan et al., 2015; Matthijs et al., 2016, 2017; Remmers et al., 2018; Smith et al., 2019), proteomes (Remmers et al., 2018), and metabolomes (Ge et al., 2014), we arranged the expression levels of related enzymes and the content of amino acids to interpret the contribution of amino acid degradation to TAG accumulation. In addition, the homologous genes and their transcription levels (Bender et al., 2014) involved in the catabolic pathways of amino acids in *Thalassiosira pseudonana* were also provided.

## Leucine, Isoleucine, and Valine

**FIGURE 1:**
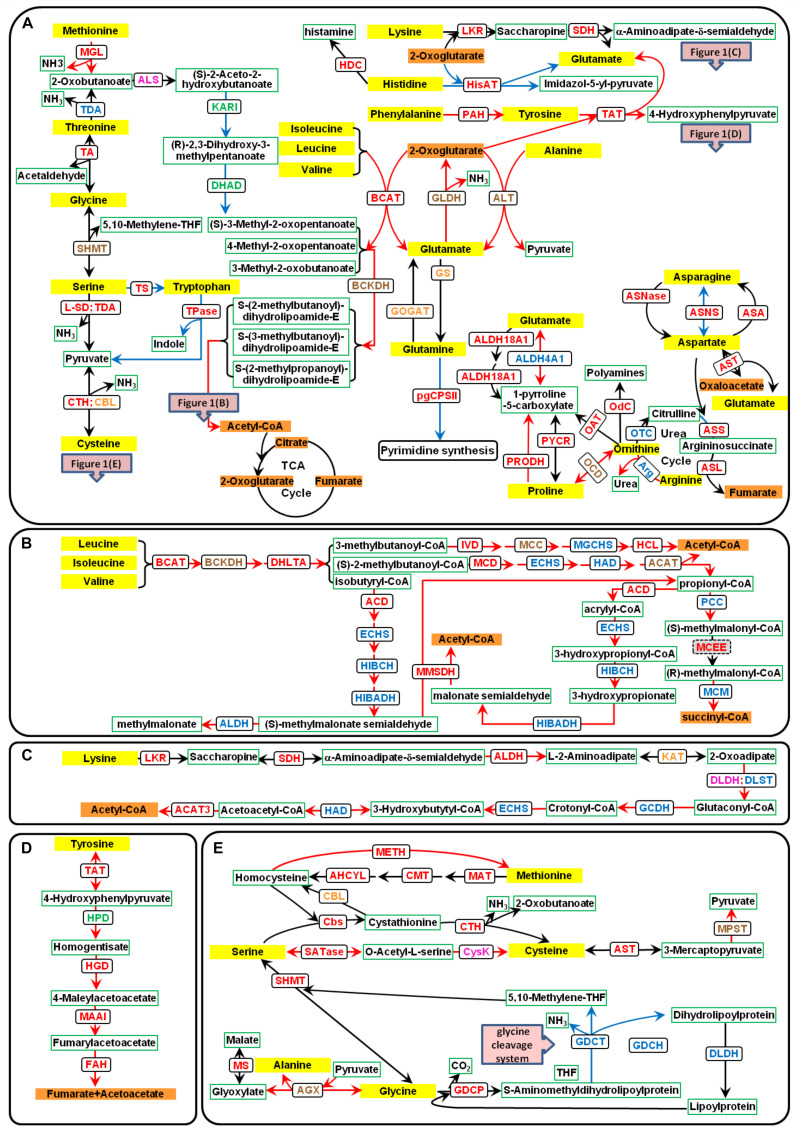
Amino acid catabolic pathways in *P. tricornutum*. The yellow text box represents amino acids, the orange text boxes indicate metabolites that could enter the TCA cycle, the white text boxes with green borders indicate other metabolites, and the white text boxes with black borders indicate the enzymes. The different font colors of enzymes represent different predicted subcellular locations: plastid localization in green font; mitochondria location in blue font; multiple isoenzymes with the plastid and mitochondria localization in orange font; those with mitochondria and other location in brown font; those with plastid and other localization in purple font; and those with plastid, mitochondria, and other localization in red font. Up-regulated genes are indicated with red lines, and down-regulated genes with blue lines. **(A)** Catabolic pathways of all 20 amino acids. **(B)** Catabolic pathways of BCAAs. **(C)** Catabolic pathways of lysine. **(D)** Catabolic pathways of tyrosine. **(E)** Catabolic pathways of serine, methionine, cysteine, and glycine. ACAT, acetyl-CoA *C*-acyltransferase (EC2.3.1.16); ACD, acyl-CoA dehydrogenase (EC1.3.8.1); AGX, alanine–glyoxylate aminotransferase (EC:2.6.1.44); AHCYL, adenosylhomocysteinase (EC3.3.1.1); ALDH, aldehyde dehydrogenase (EC1.2.1.31); ALDH18A1, delta-1-pyrroline-5-carboxylate synthetase (EC2.7.2.11 and EC1.2.1.41); ALDH4A1, 1-pyrroline-5-carboxylate dehydrogenase (EC1.2.1.88); ALS, acetolactate synthase (EC2.2.1.6); ALT, alanine transaminase (EC2.6.1.2); Arg, arginase (EC3.5.3.1); ASA, aspartate–ammonia ligase (EC6.3.1.1); ASL, argininosuccinate lyase (EC4.3.2.1); ASNase, asparaginase (EC3.5.1.1); ASNS, asparagine synthase (EC6.3.5.4); ASS, argininosuccinate synthase (EC6.3.4.5); AST, aspartate aminotransferase (EC2.6.1.1); BCAT, branched-chain amino acid transaminase (EC2.6.1.42); BCKDH, branched-chain α-keto acid dehydrogenase (EC1.2.4.4); CBL, cysteine-*S*-conjugate beta-lyase (EC4.4.1.13); Cbs, cystathionine beta-synthase (EC4.2.1.22); CMT, DNA (cytosine-5)-methyltransferase (EC2.1.1.37); CPSII, carbamoyl-phosphate synthase II (EC6.3.5.5); CTH, cystathionine gamma-lyase (EC4.4.1.1); CysK, cysteine synthase (EC2.5.1.47); DHAD, dihydroxy-acid dehydratase (EC4.2.1.9); DHLTA, dihydrolipoyllysine-residue (2-methylpropanoyl) transferase (EC2.3.1.168); DLDH, dihydrolipoyl dehydrogenase (EC1.8.1.4); DLST, dihydrolipoamide succinyltransferase; ECHS, enoyl-CoA hydratase (EC4.2.1.17); FAH, fumarylacetoacetase (EC3.7.1.2); GCDH, glutaryl-CoA dehydrogenase (EC1.3.8.6); GDCH, glycine cleavage system H protein; GDCP, glycine decarboxylase p-protein (EC1.4.4.2); GDCT, glycine decarboxylase t-protein (EC2.1.2.10); GLDH, glutamate dehydrogenase (EC1.4.1.2 and EC1.4.1.4); GOGAT, glutamine 2-oxoglutarate aminotransferase (EC1.4.1.13, EC1.4.1.14, and EC1.4.7.1); GS, glutamine synthetase (EC6.3.1.2); HAD, 3-hydroxyacyl-CoA dehydrogenase (EC1.1.1.35); HCL, hydroxymethylglutaryl-CoA lyase (EC4.1.3.4); HDC, histidine decarboxylase (EC:4.1.1.22); HGD, homogentisate 1,2-dioxygenase (EC1.13.11.5); HIBADH, 3-hydroxyisobutyrate dehydrogenase (EC1.1.1.31); HIBCH, 3-hydroxyisobutyryl-CoA hydrolase (EC3.1.2.4); HisAT, histidine transaminase (EC2.6.1.38); HPD, 4-hydroxyphenylpyruvate dioxygenase (EC1.13.11.27); IVD, isovaleryl-CoA dehydrogenase (EC1.3.8.4); KARI, ketol-acid reductoisomerase (EC1.1.1.86); KAT, kynurenine aminotransferase (EC2.6.1.39); LKR, lysine-2-oxoglutarate reductase (EC1.5.1.8); L-SD, L-serine ammonia-lyase (EC4.3.1.17); MAAI, maleylacetoacetate isomerase (EC5.2.1.2); MAT, *S*-adenosylmethionine synthetase (EC2.5.1.6); MCC, methylcrotonyl-CoA carboxylase (EC6.4.1.4); MCD, 2-methylacyl-CoA dehydrogenase (EC1.3.99.12); MCEE, methylmalonyl-CoA epimerase (EC5.1.99.1); MCM, methylmalonyl-CoA mutase (EC5.4.99.2); METH, methionine synthase (EC2.1.1.13); MGCHS, methylglutaconyl-CoA hydratase (EC4.2.1.18); MGL, methionine gamma-lyase (EC4.4.1.11); MMSDH, methylmalonate semialdehyde dehydrogenase (EC1.2.1.27); MPST, 3-mercaptopyruvate sulfurtransferase (EC2.8.1.2); MS, malate synthase (EC2.3.3.9); OAT, ornithine aminotransferase (EC2.6.1.13); OCD, ornithine cyclodeaminase (EC4.3.1.12); Odc, ornithine decarboxylase (EC4.1.1.17); OTC, ornithine carbamoyltransferase (EC2.1.3.3); PAH, phenylalanine hydroxylase (EC1.14.16.1); PCC, propionyl-CoA carboxylase (EC6.4.1.3); PRODH, proline dehydrogenase (EC1.5.5.2); PYCR, pyrroline-5-carboxylate reductase (EC1.5.1.2); SATase, serine *O*-acetyltransferase (EC2.3.1.30); SDH, saccharopine dehydrogenase (EC1.5.1.9); SHMT, serine hydroxymethyltransferase (EC2.1.2.1); TA, threonine aldolase (EC4.1.2.5); TAT, tyrosine aminotransferase (EC2.6.1.5); TDA, threonine deaminase (EC4.3.1.19); Tpase, tryptophanase (EC4.1.99.1); TS, tryptophan synthase (EC4.2.1.20)..

**FIGURE 2:**
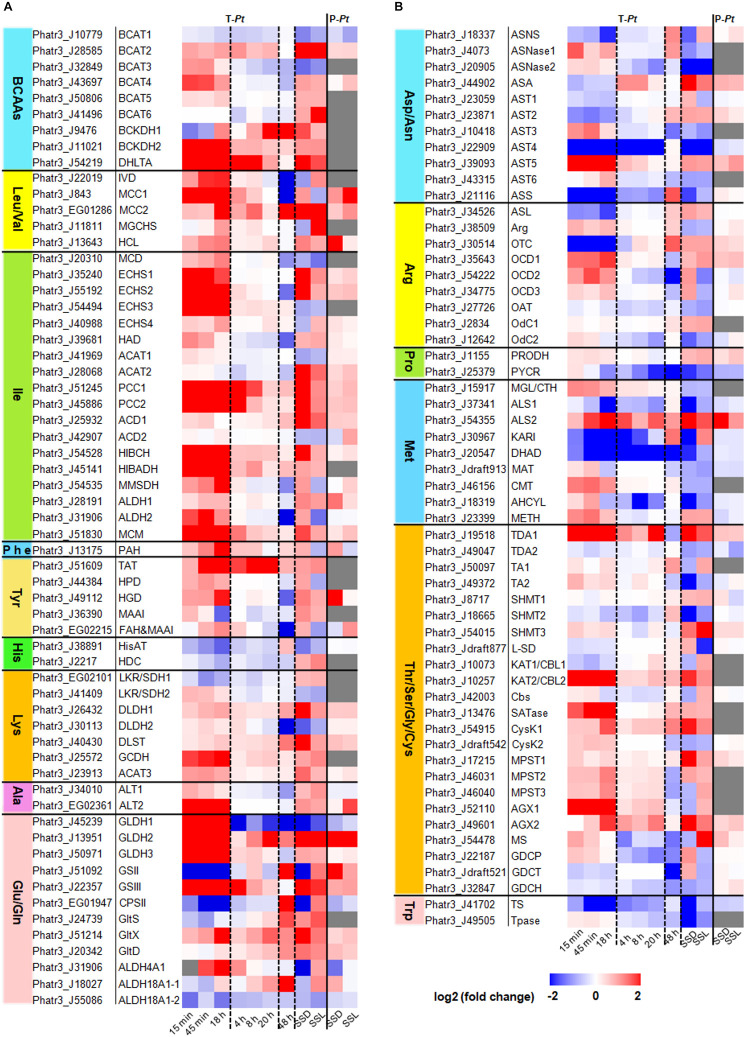
Expression levels of amino acid catabolism-related genes in *P. tricornutum*. Transcriptome data of 15 min, 45 min, and 18 h were cited from Smith et al. (2019). Fold changes of 15 min, 45 min, and 18 h were re-calculated by N-4, N-5, and N-6 contrasting with pre_3, respectively. Transcriptome data of 4, 8, and 20 h were cited from Matthijs et al. (2016, 2017). Transcriptome data of 48 h were cited from Levitan et al. (2015). The transcriptome data and the proteome data of SSL (nitrogen stress to steady state in the light period) and SSD (nitrogen stress to steady state in the dark period) were cited from Remmers et al. (2018). T-*Pt*, transcriptome of *P. tricornutum*; P-*Pt*, proteome of *P. tricornutum*. The homologous genes in *T. pseudonana* and the fold changes and RPKM data were shown in Supplementary Tables 1, 2, respectively. All the experimental conditions were shown in the Supplementary Material. The abbreviations of related enzymes are the same as those in Figure 1.

Branched-chain amino acid (leucine, valine, and isoleucine) content decreased in *P. tricornutum* cells during TAG accumulation (Supplementary Figure 1). The catabolism of BCAAs has been mostly unraveled in our previous studies (Figures 1A,B; Ge et al., 2014; Pan et al., 2017). The initial steps in the degradation pathways of BCAAs are catalyzed by branched-chain amino acid transaminase (BCAT), which also catalyzes the final step in BCAA synthesis (Figures 1A,B). Many copies of BCATs have been identified in both plants and humans. In *Arabidopsis*, there are seven isoforms of BCAT localized in different compartments, and the mitochondrial isoform BCAT2 has been shown to be especially relevant to degradation (Angelovici et al., 2013). Six *BCATs* were annotated in *P. tricornutum* genome, and all *BCATs* could be up-regulated during nitrogen limitation except *BCAT1* (Figure 2A and Supplementary Table 1). The function of BCATs depends on their localizations (Campbell et al., 2001), and thus, it is reasonable that the chloroplast-localized *P. tricornutum* BCAT1, which may be mainly responsible for BCAA synthesis, is down-regulated during nitrogen limitation. Transcriptional and/or protein levels of the other genes involved in BCAA catabolism were also found to be up-regulated during nitrogen limitation in our previous studies (Ge et al., 2014; Pan et al., 2017). The roles of methylcrotonyl-CoA carboxylase (MCC), propionyl-CoA carboxylase (PCC), 3-hydroxyisobutyryl-CoA hydrolase (HIBCH), and branched-chain α-keto acid dehydrogenase (BCKDH) in TAG accumulation have been demonstrated by genetic manipulation (Ge et al., 2014; Pan et al., 2017; Liang et al., 2019). Knockdown of *MCC* or knockout of *BCKDH* led to decreased TAG accumulation (Ge et al., 2014; Liang et al., 2019), and overexpression of *HIBCH* or knockdown of *PCC* increased TAG accumulation (Pan et al., 2017). Carbon skeletons from BCAA degradation enter the TCA cycle through acetyl-CoA in the mitochondria. The single-copy HIBCH in *P. tricornutum* has been proved to be localized in the mitochondria (Pan et al., 2017). Moreover, subcellular localization prediction shows that single-copy β-subunit of BCKDH (BCKDH2), α-subunit of MCC (MCC1), α-subunit of PCC (PCC1), 3-hydroxyisobutyrate dehydrogenase (HIBADH), and methylmalonyl-CoA mutase (MCM) together with two aldehyde dehydrogenases (ALDHs) are located in the mitochondria (Supplementary Table 1). Different from *P. tricornutum*, the whole BCAA degradation in plants takes place in both the mitochondria and peroxisome (Zolman et al., 2001).

## Histidine, Lysine, Phenylalanine, and Tyrosine

Histidine (His) is converted to glutamate (Glu) by four enzymatic steps in animals (Litwack, 2018). However, this pathway has not yet been investigated in plants. In *P. tricornutum*, there is a homologous histidine transaminase (HisAT), which could convert His to Glu and imidazol-5-yl-pyruvate using 2-oxoglutarate as a carbon skeleton (Figure 1A). The degradation pathway of imidazol-5-yl-pyruvate is not clear yet in *P. tricornutum*. Free His content was very low in *P. tricornutum* cells and was almost undetectable during nitrogen deficiency in our previous studies (Supplementary Figure 1; Ge et al., 2014; Pan et al., 2017). The expression of HisAT was down-regulated during nitrogen limitation (Figure 2A), which suggests that His might mainly be metabolized to histamine by the action of histidine decarboxylase (HDC) in the mitochondria (Figure 1A). It is likely that His degradation has no contribution to TAG accumulation.

Lysine (Lys) is catabolized *via* the α-amino adipic acid pathway identical to that in plants (Hildebrandt et al., 2015). It is converted to saccharopine and subsequently to α-aminoadipate-δ-semialdehyde by a bifunctional enzyme with two functionally independent domains, namely, lysine-ketoglutarate reductase (LKR) and saccharopine dehydrogenase (SDH), using 2-oxoglutarate as substrate to produce Glu (Figure 1C). The response of the bifunctional LKR/SDH enzyme encoding genes (*Phatr3_EG02101* and *Phatr3_J41409*) to nitrogen limitation was different between nitrogen-starved batch cultures and continuous cultures (Figure 2A). In *Arabidopsis*, the expression of LKR/SDH was regulated by abscisic acid (ABA), jasmonate, sugar starvation, and/or nitrogen starvation (Stepansky and Galili, 2003). Some enzymes in the catabolic pathways of Lys are shared by other amino acids, including enoyl-CoA hydratase (ECHS) and 3-hydroxyacyl-CoA dehydrogenase (HAD) involved in the catabolic pathways of isoleucine (Ile), and kynurenine aminotransferase (KAT, also annotated as cysteine-*S*-conjugate beta-lyase, CBL) in cysteine (Cys) metabolism (Figures 1A,E). Almost all the genes involved in Lys degradation were up-regulated with the decrease of Lys under nitrogen limitation (Figure 2A and Supplementary Figure 1). The early steps of Lys degradation (from Lys to α-aminoadipate-d-semialdehyde) may take place in the cytoplasm and the later steps (from α-aminoadipate-d-semialdehyde to acetyl-CoA) in the mitochondria according to subcellular localization prediction (Supplementary Table 1). The reaction of 2-oxoadipate oxidatively decarboxylated to glutaryl-CoA is equivalent to the oxidative decarboxylation of 2-oxoglutarate in the TCA cycle. The subsequent degradation reaction was similar to that of Ile in the mitochondria. Since one of the end degradation products of Lys is acetyl-CoA (Figure 1C) and the genes involved in Lys degradation are up-regulated, it is likely that Lys catabolism participates in the regulation of carbon/nitrogen partitioning and TAG accumulation.

Like that in animals, phenylalanine (Phe) is hydroxylated to tyrosine (Tyr) by phenylalanine hydroxylase (PAH) prior to degradation in *P. tricornutum* (Figure 1A). However, the catabolic pathway remains largely unknown since no PAH homolog has been found in plants (Hildebrandt et al., 2015). The complete degradation pathway of Tyr has been demonstrated in plants based on the degradation pathway in mammals (Dixon and Edwards, 2006). The amino group is transferred to Glu by tyrosine aminotransferase (TAT), and the product was finally degraded into fumarate and acetoacetate by four enzymatic steps. The homologous enzymes for each step have been found in *P. tricornutum* (Figure 1D). Phatr3_EG02215 annotated as fumarylacetoacetase has two annotations in UniProt, namely, fumarylacetoacetase and glutathione *S*-transferase (GST). Peptides of the latter annotation of protein Phatr3_EG02215 are homologous with human glutathione *S*-transferase zeta 1 (GSTZ1), which was also described as maleylacetoacetate isomerase (MAAI). The amino acid sequence of GST in *P. tricornutum* shares 48 and 53% identity with mammalian and *T. pseudonana* MAAI, respectively, and all the three sequences contain the conserved motif and active site of MAAI (Polekhina et al., 2001; Supplementary Figure 2). This means that Phatr3_EG02215, annotated as one gene (fumarylacetoacetase encoding gene) in *Ensembl Protists*, may actually be a compound of two genes (fumarylacetoacetase encoding gene and *MAAI*). In addition, Phatr3_J36390 was annotated as MAAI in the studies of Levering et al. (2016, 2017), though it is more similar to glutathione *S*-transferase alpha (GSTA) than GSTZ1 by BlastP analysis. The concentration of Phe and Tyr was decreased during nitrogen limitation in *P. tricornutum* (Supplementary Figure 1). The expression levels of genes involved in Phe and Tyr degradation were up-regulated accordingly (Figure 2A). Phe and Tyr catabolic pathways may take place in the cytoplasm according to subcellular localization prediction (Supplementary Table 1). Although fumarate, the final product of Phe and Tyr degradation, is an intermediate of the TCA cycle, the contribution of the catabolic pathways of the two amino acids to TAG accumulation still needs to be investigated.

## Alanine, Glutamate, and Glutamine

Alanine (Ala) can be directly converted to pyruvate by alanine aminotransferases (ALT). Glutamate dehydrogenase (GLDH) catalyzes oxidative deamination of Glu to produce 2-oxoglutarate (Figure 1A). The glutamine synthetase (GS)/glutamine 2-oxoglutarate aminotransferase (GOGAT) pathway is important for ammonium assimilation. GS catalyzes Glu and ammonium to produce glutamine (Gln), and Glu also provides α-amino group for all other amino acid biosynthesis directly or indirectly. Glu and Gln are the most important amino acids as donors for the biosynthesis of major N-containing compounds, including amino acids, nucleotides, chlorophylls, polyamines, and alkaloids (Ireland and Lea, 1999). GS is found, as multiple isoenzyme forms, located both in the cytosol (GS1) and chloroplast/plastid (GS2) and plays distinct roles in most of the higher plants (Ireland and Lea, 1999; Lancien et al., 2000). In *P. tricornutum*, one GS is located in the plastid (GSII, Phatr3_J51092) and the other one in the mitochondria (GSIII, Phatr3_J22357) (Smith et al., 2019). GOGAT transfers the amide-nitrogen of Gln to 2-oxoglutarate, thus providing two molecules of Glu (Forde and Lea, 2007). There are three forms of GOGAT in *P. tricornutum* (Alipanah et al., 2015): one that uses reduced ferredoxin as the electron donor (Fd-GOGAT/GltS, EC 1.4.7.1, Phatr3_J24739), one that uses NADH as the electron donor (NADH-GOGAT/GltX, EC 1.4.1.14, Phatr3_J51214), and the third that uses NADPH as the electron donor (NADPH-GOGAT/GltD, EC 1.4.1.13, Phatr3_J20342). The first two GOGAT present in plants are located in the chloroplast or plastid (Oliveira et al., 1997), and the third is found in bacteria (Reitzer, 1987). The subcellular localization of GOGAT in *P. tricornutum* is not the same to that in plants, which may be contributed to their different origins (Smith, 2018). GltS is located in the plastid, and GltX and GltD are predicated to be in the mitochondria. Thus, the plastidial GSII and GltS are responsible for the assimilation of ammonium produced by nitrate reduction, while mitochondrial GSIII, GltX, and GltD may catalyze the assimilation of Gln from ammonium derived from cytosolic catabolic reactions, e.g., deamination and hydrolysis of organic N (Hockin et al., 2012; Alipanah et al., 2015). pgCPSII uses Gln to perform the first committed step of pyrimidine synthesis (Allen et al., 2011). Besides, Glu can also be converted to 1-pyrroline-5-carboxylate, the intermediate product of proline (Pro) and ornithine (Orn) degradation, by one-step catalytic reaction of 1-pyrroline-5-carboxylate dehydrogenase (ALDH4A1) or by two-step catalytic reaction of delta-1-pyrroline-5-carboxylate synthetase (ALDH18A1) (Figure 1A).

The contents of Ala, Glu, and Gln dropped sharply, and Gln was even undetectable during nitrogen limitation in *P. tricornutum* (Supplementary Figure 1). The two ALT enzymes and the three GLDH enzymes were up-regulated under nitrogen-starved batch cultures or/and continuous cultures (Figure 2A). The mitochondrial GS/GOGAT was up-regulated during nitrogen limitation to assimilate ammonium derived from cytosolic catabolic reactions. The plastidial GS/GOGAT was up-regulated only under nitrogen-starved continuous cultures, suggesting that nitrate reduction was activated in plastid during nitrogen-starved continuous cultures. On the contrary, little ammonium was produced *via* nitrate reduction in plastid under nitrogen-starved batch cultures. Glu may not be used for pyrimidine synthesis during nitrogen limitation as pgCPSII was down-regulated. The mutual conversion of Glu to 1-pyrroline-5-carboxylate was mainly catalyzed by ALDH4A1 in the mitochondria due to the up-regulation of *ALDH4A1* and almost unchanged expression level of *ALDH18A1* according to the transcriptome data. In sum, based on the up-regulated expression of *ALT* gene and the products of metabolism of Ala together with the important roles of Glu and Gln in nitrogen metabolism, it seems that Ala, Glu, and Gln catabolism may contribute to TAG accumulation during nitrogen limitation.

## Aspartate, Asparagine, Arginine, and Proline

Aspartate (Asp) and asparagine (Asn) can be converted into each other by asparagine synthase (ASNS), using Gln and Glu as substrates, respectively. Besides, Asp can be converted to Asn by aspartate–ammonia ligase (ASA) with the absorption of ammonia, and Asn can be converted to Asp by asparaginase (ASNase) with the release of ammonia. Asp can be converted to 2-oxoglutarate by aspartate aminotransferase (AST), producing oxaloacetate and Glu (Figure 1A). There are six AST encoding genes in *P. tricornutum* that are predicted to be localized in various chambers of cells. Since AST was up-regulated, Asp may be converted to oxaloacetate during nitrogen limitation. The aspartate–argininosuccinate shunt established an association between the ornithine-urea cycle (OUC) and the TCA cycle (Morris, 2002; Allen et al., 2011). In addition, arginine (Arg), Orn, and Pro are directly connected to the OUC, which has been well elaborated in *P. tricornutum* (Allen et al., 2011). Although the contents of Asp, Asn, Arg, Pro, and Orn were decreased during nitrogen limitation (Supplementary Figure 1), neither the expression level of genes responsible for the conversion between Asp and Asn nor that of those genes related to OUC was significantly up-regulated (Figure 2B). Orn can be metabolized by ornithine carbamoyltransferase (OTC), ornithine decarboxylase (OdC), ornithine aminotransferase (OAT), or ornithine cyclodeaminase (OCD), and only OCD is markedly up-regulated during nitrogen limitation. Arg can be converted to Orn by arginase, which was up-regulated during nitrogen limitation. Therefore, it is very likely that the degradation of Arg produces Pro subsequently converted to 1-pyrroline-5-carboxylate by proline dehydrogenase (PRODH). Then, 1-pyrroline-5-carboxylate is converted to Glu to provide nitrogen in cells for growth. Based on the final degradation products of these four amino acids, their catabolism may contribute little to TAG accumulation.

## Methionine

The pathway for methionine (Met) degradation that converts Met to 2-oxobutanoate by methionine gamma-lyase (MGL) has been identified in plants but is absent in animals (Rébeillé et al., 2006). 2-Oxobutanoate is a precursor for Ile synthesis, and similar to Ile, it can also be degraded to acetyl-CoA *via* oxidation (Figure 1A). An alternative pathway that converts Met to homocysteine, which is subsequently converted to Cys in animals and plants, is catalyzed by three enzymes (Figure 1E). Homocysteine can be directly converted to Met, which is catalyzed by methionine synthase (METH). The expression of genes for Met degradation through 2-oxobutanoate was down-regulated, except for one acetolactate synthase (ALS) encoding gene (Figure 2B). ALS catalyzes the degradation of 2-oxobutanoate, an intermediate of threonine (Thr) catabolism. Genes involved in other Met degradation pathways were slightly up-regulated during nitrogen limitation with the decrease of Met (Supplementary Figure 1). It is not clear which pathway was dominant for the degradation of Met during nitrogen limitation in *P. tricornutum*.

## Threonine, Glycine, Serine, Cysteine, and Tryptophan

The catabolic pathways of the five amino acids are very complicated, and some involved enzymes are also present in other amino acid degradation pathways. As mentioned above, Thr can be converted to 2-oxobutanoate by threonine deaminase (TDA). It can also be interconverted with glycine (Gly) by threonine aldolase (TA). Gly can also be interconverted with serine (Ser) by serine hydroxymethyltransferase (SHMT). The easy interconversion of these three amino acids indicates that these reactions can be relevant for the synthesis of the product amino acids and the degradation of the substrate amino acids as well (Figure 1A; Hildebrandt et al., 2015).

Cysteine and tryptophan (Trp) can be produced from Ser by two and one enzymatic reactions, respectively (Figure 1A). The conversion of the former can be completed through the intermediate product cystathionine or *O*-acetyl-L-serine (Figure 1E). Ser, Trp, and Cys can be directly degraded into pyruvate with the release of ammonia or indole. In addition, Cys can also be degraded into pyruvate through a two-step reaction (Hildebrandt et al., 2015; Figure 1E). The Gly cleavage system (GCS), which is a mitochondrial multi-enzyme system (also named glycine decarboxylase or glycine dehydrogenase system), comprises four proteins, three enzymes (P-protein, T-protein, and L-protein), and a small lipoylated protein known as H-protein. GCS, an essential and ubiquitous step of both photorespiration and primary metabolism in plants, is responsible for the interconversion of Gly and Ser (Bauwe and Kolukisaoglu, 2003). Although the H-protein has no catalytic activity itself, it acts as a substrate for the P-, T-, and L-proteins and increases the GCS activity (Hasse et al., 2009). Gly can also be transaminated by alanine–glyoxylate aminotransferase (AGX), and the resulting glyoxylate can be acetylated in the peroxisomes to produce malate in plants (Mazelis, 1980). Homologs of genes mentioned above are present in the *P. tricornutum* genome (Figure 1E), although the locations of the encoded proteins are not exactly the same as those in plants (Supplementary Table 1). Thr and Trp contents decreased and Ser content showed no marked difference, while Gly content increased during nitrogen limitation in *P. tricornutum* (Supplementary Figure 1). Cys was not detected in our previous studies (Ge et al., 2014; Pan et al., 2017). In general, Thr and Trp may be degraded mainly through the catalysis of TDA and tryptophanase (Tpase), respectively. The conversion of Ser to Cys and the degradation of Cys may be active during nitrogen limitation according to gene expression levels (Figure 2B). However, Ser might not be converted to Trp but was interconverted with Gly frequently. Since GCS-related genes were not up-regulated during nitrogen limitation, Gly was not degraded mainly by GCS. Up-regulated AGX and malate synthase (MS) indicated that Gly was transaminated and acetylated under nitrogen starvation, which may help to explain why the content of Gly did not decrease and even increased during nitrogen limitation. In brief, the contribution of the degradation of these five amino acids to TAG accumulation remains unclear.

## Conclusion

Amino acids, as protein constituents and essential metabolites, play critical roles in living organisms. Some amino acids (e.g., serine, proline, and leucine) have been shown to act as signaling molecules in plants (Szabados and Savouré, 2010; Häusler et al., 2014; Ros et al., 2014). Therefore, pool sizes of amino acids are of critical importance and are adjusted by amino acid catabolism. The degradation pathways of amino acids in *P. tricornutum* were not identical with those in plants and mammals. In particular, the metabolic pathway of BCAAs in the diatom is different from that in animals, and the subcellular locations of related enzymes are not exactly the same with those in plants. In addition, the metabolic pathways of His and Phe and the OUC in *P. tricornutum* are similar to those in animals, but no related enzymes are found in plants. The mutual transformation pathway of essential amino acids in *P. tricornutum* does not exist in animals. Considering the end degradation products of amino acids and the expression levels of related enzymes in the metabolic pathways during nitrogen limitation, BCAAs, Lys, Ala, Glu, and Gln may contribute to TAG accumulation. Furthermore, to fully understand the catabolic pathways and their regulatory mechanisms, genetic manipulation and a combination of post-genomic approaches (transcriptome, proteome, and metabolome) are necessary for the analyses of mutant and wild-type diatoms.

## Author Contributions

YP, CY, CL, and TH arranged the data under the guidance of HH. YP and HH prepared and wrote the manuscript. FH reviewed and revised this manuscript. All authors contributed to the article and approved the submitted version.

## Conflict of Interest

The authors declare that the research was conducted in the absence of any commercial or financial relationships that could be construed as a potential conflict of interest.

## References

[B1] Admiraal W. Peletier H. (1979). Influence of organic compounds and light limitation on the growth rate of estuarine benthic diatoms. *Br. Phycol. J.* 14 197–206. 10.1080/00071617900650211

[B2] Alipanah L. Rohloff J. Winge P. Bones A. M. Brembu T. (2015). Whole-cell response to nitrogen deprivation in the diatom *Phaeodactylum tricornutum*. *J. Exp. Bot.* 66 6281–6296. 10.1093/jxb/erv340 26163699 PMC4588885

[B3] Allen A. E. Dupont C. L. Oborník M. Horák A. Nunes-Nesi A. McCrow J. P. (2011). Evolution and metabolic significance of the urea cycle in photosynthetic diatoms. *Nature* 473 203–207. 10.1038/nature10074 21562560

[B4] Angelovici R. Lipka A. E. Deason N. Gonzalez-Jorge S. Lin H. Cepela J. (2013). Genome-wide analysis of branched-chain amino acid levels in *Arabidopsis* seeds. *Plant Cell* 25 4827–4843. 10.1105/tpc.113.119370 24368787 PMC3903990

[B5] Armbrust E. V. Berges J. A. Bowler C. Green B. R. Martinez D. Putnam N. H. (2004). The genome of the diatom *Thalassiosira pseudonana*: ecology, evolution, and metabolism. *Science* 306 79–86. 10.1126/science.1101156 15459382

[B6] Bauwe H. Kolukisaoglu Ü. (2003). Genetic manipulation of glycine decarboxylation. *J. Exp. Bot.* 54 1523–1535. 10.1093/jxb/erg171 12730263

[B7] Bender S. J. Durkin C. A. Berthiaume C. T. Morales R. L. Armbrust E. (2014). Transcriptional responses of three model diatoms to nitrate limitation of growth. *Front. Mar. Sci.* 1:3 10.3389/fmars.2014.00003

[B8] Bowler C. Allen A. E. Badger J. H. Grimwood J. Jabbari K. Kuo A. (2008). The *Phaeodactylum* genome reveals the evolutionary history of diatom genomes. *Nature* 456 239–244. 10.1038/nature07410 18923393

[B9] Bromke M. A. (2013). Amino acid biosynthesis pathways in diatoms. *Metabolites* 3 294–311. 10.3390/metabo3020294 24957993 PMC3901274

[B10] Butler T. Kapoore R. V. Vaidyanathan S. (2020). *Phaeodactylum tricornutum*: a diatom cell factory. *Trends Biotechnol.* 38 606–622. 10.1016/j.tibtech.2019.12.023 31980300

[B11] Campbell M. A. Patel J. K. Meyers J. L. Myrick L. C. Gustin J. L. (2001). Genes encoding for branched-chain amino acid aminotransferase are differentially expressed in plants. *Plant Physiol. Biochem.* 39 855–860. 10.1016/S0981-9428(01)01306-7

[B12] Contreras J. A. Gillard J. T. (2020). Asparagine-based production of hydrogen peroxide triggers cell death in the diatom *Phaeodactylum tricornutum*. *Bot. Lett.* 1–12. 10.1080/23818107.2020.1754289

[B13] Dixon D. P. Edwards R. (2006). Enzymes of tyrosine catabolism in *Arabidopsis thaliana*. *Plant Sci.* 171 360–366. 10.1016/j.plantsci.2006.04.008 22980205

[B14] Fabris M. Matthijs M. Rombauts S. Vyverman W. Goossens A. Baart G. J. (2012). The metabolic blueprint of Phaeodactylum tricornutum reveals a eukaryotic Entner–Doudoroff glycolytic pathway. *Plant J.* 70 1004–1014. 10.1111/j.1365-313X.2012.04941.x 22332784

[B15] Falciatore A. Jaubert M. Bouly J. P. Bailleul B. Mock T. (2020). Diatom molecular research comes of age: model species for studying phytoplankton biology and diversity. *Plant Cell* 32 547–572. 10.1105/tpc.19.00158 31852772 PMC7054031

[B16] Forde B. G. Lea P. J. (2007). Glutamate in plants: metabolism, regulation, and signalling. *J. Exp. Bot.* 58 2339–2358. 10.1093/jxb/erm121 17578865

[B17] Ge F. Huang W. Chen Z. Zhang C. Xiong Q. Bowler C. (2014). Methylcrotonyl-CoA carboxylase regulates triacylglycerol accumulation in the model diatom *Phaeodactylum tricornutum*. *Plant Cell* 26 1681–1697. 10.1105/tpc.114.124982 24769481 PMC4036579

[B18] Geider R. La Roche J. (2002). Redfield revisited: variability of C:N:P in marine microalgae and its biochemical basis. *Eur. J. Phycol.* 37 1–17. 10.1017/S0967026201003456

[B19] Guerra L. T. Levitan O. Frada M. J. Sun J. S. Falkowski P. G. Dismukes G. C. (2013). Regulatory branch points affecting protein and lipid biosynthesis in the diatom *Phaeodactylum tricornutum*. *Biomass Bioenerg.* 59 306–315. 10.1016/j.biombioe.2013.10.007

[B20] Hasse D. Mikkat S. Hagemann M. Bauwe H. (2009). Alternative splicing produces an H-protein with better substrate properties for the P-protein of glycine decarboxylase. *FEBS J.* 276 6985–6991. 10.1111/j.1742-4658.2009.07406.x 19860829

[B21] Häusler R. E. Ludewig F. Krueger S. (2014). Amino acids – A life between metabolism and signaling. *Plant Sci.* 229 225–237. 10.1016/j.plantsci.2014.09.011 25443849

[B22] Hildebrandt T. M. Nunes Nesi A. Araújo W. L. Braun H. P. (2015). Amino acid catabolism in plants. *Mol. Plant* 8 1563–1579. 10.1016/j.molp.2015.09.005 26384576

[B23] Hockin N. L. Mock T. Mulholland F. Kopriva S. Malin G. (2012). The response of diatom central carbon metabolism to nitrogen starvation is different from that of green algae and higher plants. *Plant Physiol.* 158 299–312. 10.1104/pp.111.184333 22065419 PMC3252072

[B24] Ireland R. J. Lea P. J. (1999). “The enzymes of glutamine, glutamate, asparagine, and aspartate metabolism,” in *Plant Amino Acids. Biochemistry and Biotechnology*, ed. Singh B. K. (New York, NY: Marcel Dekker), 49–109.

[B25] Jauffrais T. Jesus B. Méléder V. Turpin V. Arnaldo D. Raimbault P. (2016). Physiological and photophysiological responses of the benthic diatom *Entomoneis paludosa* (Bacillariophyceae) to dissolved inorganic and organic nitrogen in culture. *Mar. Biol.* 163:115 10.1007/s00227-016-2888-9

[B26] Karas B. J. Diner R. E. Lefebvre S. C. McQuaid J. Phillips A. P. Noddings C. M. (2015). Designer diatom episomes delivered by bacterial conjugation. *Nat. Commun.* 6:6925. 10.1038/ncomms7925 25897682 PMC4411287

[B27] Kudela R. M. Dugdale R. C. (2000). Nutrient regulation of phytoplankton productivity in Monterey Bay, California. *Deep Sea Res. Part II Top. Stud. Oceanogr.* 47 1023–1053. 10.1016/S0967-0645(99)00135-6

[B28] Lancien M. Gadal P. Hodges M. (2000). Enzyme redundancy and the importance of 2-oxoglutarate in higher plant ammonium assimilation. *Plant Physiol.* 123 817–824. 10.1104/pp.123.3.817 10889231 PMC1539263

[B29] Levering J. Broddrick J. Dupont C. L. Peers G. Beeri K. Mayers J. (2016). Genome-scale model reveals metabolic basis of biomass partitioning in a model diatom. *PLoS One* 11:e0155038. 10.1371/journal.pone.0155038 27152931 PMC4859558

[B30] Levering J. Dupont C. L. Allen A. E. Palsson B. O. Zengler K. (2017). Integrated regulatory and metabolic networks of the marine diatom *Phaeodactylum tricornutum* predict the response to rising CO2 levels. *mSystems* 2:e00142-16. 10.1128/mSystems.00142-16 28217746 PMC5309336

[B31] Levitan O. Dinamarca J. Zelzion E. Lun D. S. Guerra L. T. Kim M. K. (2015). Remodeling of intermediate metabolism in the diatom *Phaeodactylum tricornutum* under nitrogen stress. *Proc. Natl. Acad. Sci. U.S.A.* 112 412–417. 10.1073/pnas.1419818112 25548193 PMC4299248

[B32] Liang Y. Kong F. Torres-Romero I. Burlacot A. Cuine S. Legeret B. (2019). Branched-chain amino acid catabolism impacts triacylglycerol homeostasis in *Chlamydomonas reinhardtii*. *Plant Physiol.* 179 1502–1514. 10.1104/pp.18.01584 30728273 PMC6446750

[B33] Litwack G. (ed.) (2018). “Metabolism of amino acids,” in *Human Biochemistry*, (Boston, MA: Academic Press), 359–394.

[B34] Matthijs M. Fabris M. Broos S. Vyverman W. Goossens A. (2016). Profiling of the early nitrogen stress response in the diatom *Phaeodactylum tricornutum* reveals a novel family of RING-domain transcription factors. *Plant Physiol.* 170 489–498. 10.1104/pp.15.01300 26582725 PMC4704581

[B35] Matthijs M. Fabris M. Obata T. Foubert I. Franco-Zorrilla J. M. Solano R. (2017). The transcription factor bZIP14 regulates the TCA cycle in the diatom *Phaeodactylum tricornutum*. *EMBO J.* 36 1559–1576. 10.15252/embj.201696392 28420744 PMC5452028

[B36] Mazelis M. (1980). “15 - Amino acid catabolism,” in *Amino Acids and Derivatives*, ed. Miflin B. J. (Cambridge, MA: Academic Press), 541–567. 10.1016/b978-0-12-675405-6.50021-8

[B37] Medlin L. Kooistra W. Schmid A. M. (2000). “A review of the evolution of the diatoms-a total approach using molecules, morphology and geology,” in *The Origin and Early Evolution of the Diatoms: Fossil, Molecular and Biogeographical Approaches*, eds Witkowski A. Sieminska J. (Krakow: Polish Academy of Sciences), 13–35.

[B38] Moore C. M. Mills M. M. Arrigo K. R. Berman-Frank I. Bopp L. Boyd P. W. (2013). Processes and patterns of oceanic nutrient limitation. *Nat. Geosci.* 6 701–710. 10.1038/ngeo1765

[B39] Morris S. M. (2002). Regulation of enzymes of the urea cycle and arginine metabolism. *Annu. Rev. Nutr.* 22 87–105. 10.1146/annurev.nutr.22.110801.140547 12055339

[B40] Moustafa A. Beszteri B. Maier U. G. Bowler C. Valentin K. Bhattacharya D. (2009). Genomic footprints of a cryptic plastid endosymbiosis in diatoms. *Science* 324 1724–1726. 10.1126/science.1172983 19556510

[B41] Oliveira I. C. Lam H. M. Coschigano K. Melo-Oliveira R. Coruzzi G. (1997). Molecular-genetic dissection of ammonium assimilation in *Arabidopsis thaliana*. *Plant Physiol. Biochem.* 35 185–198.

[B42] Palenik B. Morel F. M. (1990). Comparison of cell-surface L-amino acid oxidases from several marine phytoplankton. *Mar. Ecol. Prog. Ser.* 59 195–201. 10.3354/meps059195

[B43] Pan Y. Yang J. Gong Y. Li X. Hu H. (2017). 3-Hydroxyisobutyryl-CoA hydrolase involved in isoleucine catabolism regulates triacylglycerol accumulation in *Phaeodactylum tricornutum*. *Philos. Trans. R. Soc. Lond. B Biol. Sci.* 372:20160409. 10.1098/rstb.2016.0409 28717019 PMC5516118

[B44] Polekhina G. Board P. G. Blackburn A. C. Parker M. W. (2001). Crystal structure of maleylacetoacetate isomerase/glutathione transferase zeta reveals the molecular basis for its remarkable catalytic promiscuity. *Biochemistry* 40 1567–1576. 10.1021/bi002249z 11327815

[B45] Rébeillé F. Jabrin S. Bligny R. Loizeau K. Gambonnet B. Van Wilder V. (2006). Methionine catabolism in *Arabidopsis* cells is initiated by a γ-cleavage process and leads to S-methylcysteine and isoleucine syntheses. *Proc. Natl. Acad. Sci. U.S.A.* 103 15687–15692. 10.1073/pnas.0606195103 17030798 PMC1622882

[B46] Rees T. A. V. Allison V. J. (2006). Evidence for an extracellular L–amino acid oxidase in nitrogen-deprived *Phaeodactylum tricornutum* (Bacillariophyceae) and inhibition of enzyme activity by dissolved inorganic carbon. *Phycologia* 45 337–342. 10.2216/04-92.1

[B47] Reitzer L. J. (1987). “Ammonia assimilation and the biosynthesis of glutamine, glutamate, aspartate, asparagine, L-alanine and D-alanine,” in *Escherichia Coli and Salmonella typhimurium. Cellular and Molecular Biology*, Vol. 2 eds Neidhardt F. C. Ingraham J. L. Low K. B. Magasanik B. Schaechter M. Umbarger H. E. (Washington, DC: American Society for Microbiology), 302–320.

[B48] Remmers I. M. D’Adamo S. Martens D. E. de Vos R. C. Mumm R. America A. H. (2018). Orchestration of transcriptome, proteome and metabolome in the diatom *Phaeodactylum tricornutum* during nitrogen limitation. *Algal Res.* 35 33–49. 10.1016/j.algal.2018.08.012

[B49] Ros R. Muñoz-Bertomeu J. Krueger S. (2014). Serine in plants: biosynthesis, metabolism, and functions. *Trends Plant Sci.* 19 564–569. 10.1016/j.tplants.2014.06.003 24999240

[B50] Singh D. Carlson R. Fell D. Poolman M. (2015). Modelling metabolism of the diatom *Phaeodactylum tricornutum*. *Biochem. Soc. Trans.* 43 1182–1186. 10.1042/BST20150152 26614658

[B51] Sipler R. E. Bronk D. A. (2015). “Dynamics of dissolved organic nitrogen,” in *Biogeochemistry of Marine Dissolved Organic Matter*, ed. Carlson D. A. H. A. (Boston, MA: Academic Press), 127–232. 10.1016/B978-0-12-405940-5.00004-2

[B52] Smith S. (2018). *Figshare: Nitrogen Gene Phylogenies.* Available online at: 10.6084/m9.figshare.6233198 (accessed October 15, 2020).

[B53] Smith S. R. Dupont C. L. McCarthy J. K. Broddrick J. T. Oborník M. Horák A. (2019). Evolution and regulation of nitrogen flux through compartmentalized metabolic networks in a marine diatom. *Nat. Commun.* 10:4552. 10.1038/s41467-019-12407-y 31591397 PMC6779911

[B54] Stepansky A. Galili G. (2003). Synthesis of the *Arabidopsis* bifunctional lysine-ketoglutarate reductase/saccharopine dehydrogenase enzyme of lysine catabolism is concertedly regulated by metabolic and stress-associated signals. *Plant Physiol.* 133 1407–1415. 10.1104/pp.103.026294 14576281 PMC281635

[B55] Szabados L. Savouré A. (2010). Proline: a multifunctional amino acid. *Trends Plant Sci.* 15 89–97. 10.1016/j.tplants.2009.11.009 20036181

[B56] Zhang C. Hu H. (2014). High-efficiency nuclear transformation of the diatom *Phaeodactylum tricornutum* by electroporation. *Mar. Genomics* 16 63–66. 10.1016/j.margen.2013.10.003 24269346

[B57] Zolman B. K. Monroe-Augustus M. Thompson B. Hawes J. W. Krukenberg K. A. Matsuda S. P. (2001). chy1, an Arabidopsis mutant with impaired β-oxidation, is defective in a peroxisomal β-hydroxyisobutyryl-CoA hydrolase. *J. Biol. Chem.* 276 31037–31046. 10.1074/jbc.M104679200 11404361

