# Supplementary material for: Amino Acid Catabolism During Nitrogen Limitation in Phaeodactylum tricornutum
Source: Front Plant Sci. 2020 Dec 17;11:589026. doi: 10.3389/fpls.2020.589026 (PMC7780933; doi:10.3389/fpls.2020.589026)
Supplement: Supplementary file 1 [file Data_Sheet_1.pdf]

1. **Figure S1. Amino acid contents in *P. tricornutum* grown in f/2 ($NaNO_3$ concentration was reduced to 500 μM) enriched artificial seawater medium. Error bars represent SE of three biological replicates (Data from Ge et al., 2014).**

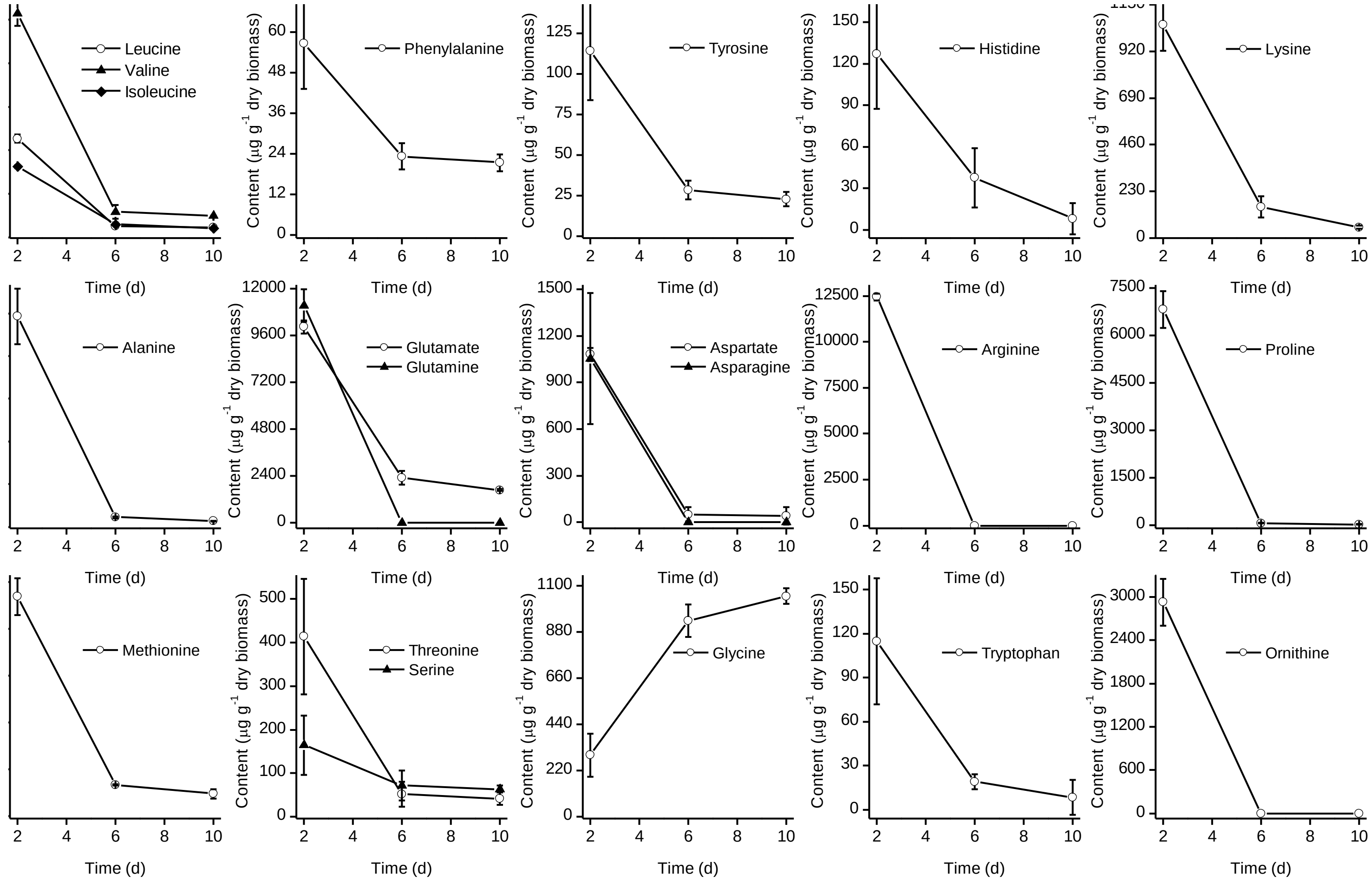

Nitrogen was limited after 6 days and then triacylglycerols (TAGs) accumulated.

Reference

Ge, F., Huang, W., Chen, Z., Zhang, C., Xiong, Q., Bowler, C. et al. (2014). Methylcrotonyl-CoA carboxylase regulates triacylglycerol accumulation in the model diatom *Phaeodactylum tricornutum*. *Plant Cell*, 26, 1681-1697. doi: 10.1105/tpc.114.124982

2. Growth and experiments conditions in Figure 2

(1) Transcriptome data of 15 min, 45 min and 18 h (nitrogen starved batch cultures) (Smith et al., 2019)

Duplicate 2 L cultures of *P. tricornutum* (CCAP-1055) were grown on artificial seawater medium with f/2 nutrients, trace metals, and vitamins with 880 μM ammonium as the sole nitrogen source and were stirred and bubbled with air with 14:10 light:dark (150 μE m^{-2} s^{-1}) at 18 ℃. Cells were collected by centrifugation at mid-exponential phase (~3×10^{6} cells mL^{-1}), washed, and resuspended in N-free media in respective flasks (2 L) for 2 h. Replicate cultures were spiked with nitrate to 150 μM and incubated for 90 min. Cells were collected by centrifugation, washed, and material from each replicate independently split into 800 mL no nitrogen treatments. Samples were taken during the pretreatment (at mid-exponential (pre_3), and after the 2-h N-pretreatment (pre_2) and 90 min nitrate incubations (pre_3)) and at 15 min (N-5), 45 min (N-4), and 18 h (N-6) exposure to no nitrogen treatments. Fold changes of 15 min, 45 min and 18 h were re-calculated by N-4, N-5 and N-6 contrasting with pre_3, respectively.

(2) Transcriptome data of 4 h, 8 h and 20 h (nitrogen starved batch cultures) (Matthijs et al., 2016; Matthijs et al., 2017)

P. tricornutum (Pt1) Strain 8.6 cells were grown in 500 mL Erlenmeyer flasks with ESAW medium containing 7.5 mg sodium nitrate per liter and other nutrients. Flasks were placed on a shaking platform at 120 rpm with an average lighting intensity of 100 μE m^{-2} s^{-1} in a temperature controlled room at 21 ℃. Exponentially growing *P. tricornutum* cells were transferred to nitrogen-replete medium, and sampled 4, 8, and 20 h after medium transfer.

(3) Transcriptome data of 48 h (nitrogen starved batch cultures) (Levitan et al., 2015)

P. tricornutum (accession Pt1 8.6) was maintained axenically in sterile artificial seawater enriched with F/2 nutrients. Three independent cultures was maintained under exponential growth conditions starting at 2.5×10^5 cells/mL in flasks at 18 ℃ and 120–150 μmol photons m^{-2} s^{-1} continuous white light emitting diodes and aerated through 0.2-μm filters. After 48 h of growth, cells were centrifuged, washed two times with nitrogen-free, artificial seawater-based F/2, and split into nitrogen-replete and -free conditions. To assure the largest contrast between the physiological states, both treatments were sampled after 48 h.

(4) Transcriptome data and proteome data of SSL and SSD (nitrogen starved continuous cultures) (Remmers et al., 2018)

P. tricornutum SAG1090-1b cells cultivated aseptically in a flat panel airlift-loop reactor with a working volume of 1.7 L and a light path of 0.02 m (Labfors 5 Lux, Infors HT, Switzerland). Cultures were continuously purged with 1.7 L min^{-1} air enriched with 1% CO_2. The temperature was controlled at 20 ℃ and the pH was maintained at 7.2 using 5% H_2SO_4. 1–2 drops of 1% w/w antifoam (Antifoam B, Baker, the Netherlands) were added once per day. The culture was exposed to two feeding regimes using a separate nitrogen (N) feed: N replete growth (control experiment, nitrogen supply rate of 0.11 g N day^{-1}) and N limited growth (0.02 g N day^{-1}). When the culture reached steady state, samples were taken at 5–6 h intervals for biomass composition, proteome, metabolome and transcriptom analysis. All

samples taken in the light period are separated from samples taken after the 8 h of darkness.

(5) Transcriptome data and proteome data of *T. pseudonana* (nitrogen starved continuous cultures) (Bender et al., 2014)

Thalassiosira pseudonana were maintained without bubbling in semi-continuous batch cultures under continuous light (100 μmol photons m^{-2} s^{-1}) in modified artificial seawater with f/2 concentrations of nutrients (882 μM $NaNO_3$, 106 μM Na_2SiO_3, 36.2 μM NaH_2PO_4) at 20 ℃. Cultures were considered acclimated to these growth conditions when the growth rates of three consecutive transfers were not significantly different from one another. Each acclimated diatom culture was then transferred into a batch culture with nutrient-replete media (882 μM $NaNO_3$, 10 6μM Na_2SiO_3, 36.2 μM NaH_2PO_4) and a batch culture with low nitrate media (55 μM $NaNO_3$, 212 μM Na_2SiO_3, 72.4 μM NaH_2PO_4) in artificial seawater, maintaining three biological replicates per condition. Experiments were conducted in 10 L bottles; all cultures were bubbled with sterile filtered air. The experimental cultures were filtered onto 0.8 μm polycarbonate filters during mid-exponential growth (nutrient-replete) or at the onset of stationary phase due to nitrate limitation.

Reference

Levitan, O., Dinamarca, J., Zelzion, E., Lun, D. S., Guerra, L. T., Kim, M. K. et al. (2015). Remodeling of intermediate metabolism in the diatom *Phaeodactylum tricornutum* under nitrogen stress. *Proc. Natl. Acad. Sci. USA*, 112, 412-417. doi: 10.1073/pnas.1419818112

Matthijs, M., Fabris, M., Broos, S., Vyverman, W., Goossens, A. (2016). Profiling of the early nitrogen stress response in the diatom *Phaeodactylum tricornutum* reveals a novel family of RING-domain transcription factors. *Plant Physiol.*, 170, 489-498. doi: 10.1104/pp.15.01300

Matthijs, M., Fabris, M., Obata, T., Foubert, I., Franco-Zorrilla, J. M., Solano, R. et al. (2017). The transcription factor bZIP14 regulates the TCA cycle in the diatom *Phaeodactylum tricornutum*. *EMBO J.*, 36(11), 1559-1576. doi: 10.15252/embj.201696392

Remmers, I. M., D'Adamo, S., Martens, D. E., de Vos, R. C., Mumm, R., America, A. H. et al. (2018). Orchestration of transcriptome, proteome and metabolome in the diatom *Phaeodactylum tricornutum* during nitrogen limitation. *Algal Res.*, 35, 33-49. doi: 10.1016/j.algal.2018.08.012

Smith, S. R., Dupont, C. L., McCarthy, J. K., Broddrick, J. T., Oborník, M., Horák, A. et al. (2019). Evolution and regulation of nitrogen flux through compartmentalized metabolic networks in a marine diatom. *Nat.Commun.*, 10, 1-14. doi: 10.1038/s41467-019-12407-y

Bender, S. J., Durkin, C. A., Berthiaume, C. T., Morales, R. L., Armbrust, E. (2014). Transcriptional responses of three model diatoms to nitrate limitation of growth. *Front. Mar. Sci.*, 1, 3. doi: 10.3389/fmars.2014.00003

3. Gene annotation and subcellular localization prediction

Functions of predicted genes were based on the annotations, which were summarized from EnsemblProtist (https://protists.ensembl.org/Phaeodactylum_tricornutum/), NCBI (https://www.ncbi.nlm.nih.gov/), Uniprot (https://www.ebi.ac.uk/uniprot/) and KEGG (https://www.kegg.jp/kegg/).

To predict a subcellular localization for each protein, we used the updated Phatr3 (https://protists.ensembl.org/Phaeodactylum_tricornutum/) protein sequences as input for TMHMM 2.0 (Krogh et al., 2001), TargetP 2.0 (Emanuelsson et al., 2000) and HECTAR (Gschloessl et al., 2008). All programs were run using default settings.

Reference

Krogh, A., Larsson, B., Von Heijne, G., Sonnhammer, E. L. (2001). Predicting transmembrane protein topology with a hidden Markov model: application to complete genomes. *J. Mol. Biol.*, 305(3), 567-580. doi: 10.1006/jmbi.2000.4315

Emanuelsson, O., Nielsen, H., Brunak, S., Von Heijne, G. (2000). Predicting subcellular localization of proteins based on their N-terminal amino acid sequence. *J. Mol. Biol.*, 300(4), 1005-1016. doi: 10.1006/jmbi.2000.3903

Gschloessl, B., Guermeur, Y., Cock, J. M. (2008). HECTAR: a method to predict subcellular targeting in heterokonts. *BMC bioinformatics*, 9(1), 393. doi: 10.1186/1471-2105-9-393

4. **Figure S2. Sequence alignment of the amino acids of glutathione S-transferase in *P. tricornutum* (Phatr3_J36390 and Uniprot: B7GDK0), *T. pseudonana* (Tp_33717), and human (GSTZ1). The conserved motif (LYSYWR/LSSCSXR/KVRIAL) and catalytic sites of maleylacetoacetate isomerase (MAAI) are indicated by red frame and black arrows, respectively.**

```
                               10        20        30        40        50        60
                       ....|....|....|....|....|....|....|....|....|....|....|....|
Hs_GSTZ1           1   MQAGKPILYSYFRSSCSWRVRIALALKGIDYETVPINLIKDGGQQFSKDFQALNPMKQVP 60
Uniprot:B7GDK0     1   ---QSFKLYGYWRSSSTWRVRIALAVKHIEYETIPIDLAGD--QQKSEGYLEVNPFGQVP 55
Tp_33717           1   -------LYGYWRSSCTWRVRIALAAKGIQYKTVPVDVYNR--DQTANEY--ANPMKQVP 49
Phatr3_J36390      1   ----MPTLYHIPKTISSPIVQCLLELDVVGNGVDVYETNFQ--EIKTETFLLRNPMGTSP 54

                               70        80        90       100       110       120
                       ....|....|....|....|....|....|....|....|....|....|....|....|
Hs_GSTZ1          61   TLKID------GITIHQSLAIIEYLEETRP----------TPRLLPQDPKKRASVRMISD 104
Uniprot:B7GDK0    56   LLECSDVLTGGTIRLGQSIAIIDFLEEAFP-L--------RKSLLPKNLVERAIARQMAE 106
Tp_33717          50   TLEFVEGGK-TVVRISQSLAIIEFLESAFADC--------GGRLLPLDPITRAKVKEIAE 100
Phatr3_J36390     55   TLEDD------NIVLWESGAVLDYLLERYDTENRLAPRCLSAESLPEHSALRAKYLHVKQ 108

                              130       140       150       160       170       180
                       ....|....|....|....|....|....|....|....|....|....|....|....|
Hs_GSTZ1         105   LIAGGIQPLQNLSVLKQVG----EEMQLTWAQNAITCGFNALEQILQS------TAGIYC 154
Uniprot:B7GDK0   107   IINSGTQPLQNIFSLRDFE----------------------------------------- 125
Tp_33717         101   IVNSGTQPLQNVRVLNALNKLAGAGYGEEFGKDGITKGLASIEQLLSPYHSEHCGAGSFA 160
Phatr3_J36390    109   YIIATVYPFVASLFLHTQR------SIEEQDEAYIQSTKRKWTSVMAPVLSQWLGEGTYF 162

                              190       200       210       220       230       240
                       ....|....|....|....|....|....|....|....|....|....|....|....|
Hs_GSTZ1         155   VG-DEVTMADLCLVPQVANAERFKVDLTP-YPTISSINKRLLVLEAFQVSHPCRQPDTPT 212
Uniprot:B7GDK0   125   ------------------------------------------------------------ 125
Tp_33717         161   TGSFGPTLADVCLVPQLYNARLFGVDVESLFPTLLKIDAVCNAHPWFHTTHPTLQIDAKI 220
Phatr3_J36390    163   LG-DQMTAVDFLVCKPLNNAN--SLGMLVVFPRLKSLLDRMKCRPTFRLAYEALPPNEES 219

                              250       260
                       ....|....|....|....|....
Hs_GSTZ1         213   ELRA-------------------- 216
Uniprot:B7GDK0   125   ------------------------ 125
Tp_33717         220   ------------------------ 220
Phatr3_J36390    220   HYNRSILVVPGMTTCQVSVEDSKA 243
```
